# Linking discoveries, mechanisms, and technologies to develop a clearer perspective on plant long noncoding RNAs

**DOI:** 10.1093/plcell/koad027

**Published:** 2023-02-04

**Authors:** Kyle Palos, Li’ang Yu, Caylyn E Railey, Anna C Nelson Dittrich, Andrew D L Nelson

**Affiliations:** Boyce Thompson Institute, Cornell University, Ithaca, NY 14853, USA; Boyce Thompson Institute, Cornell University, Ithaca, NY 14853, USA; Boyce Thompson Institute, Cornell University, Ithaca, NY 14853, USA; Plant Biology Graduate Field, Cornell University, Ithaca, NY 14853, USA; Boyce Thompson Institute, Cornell University, Ithaca, NY 14853, USA; Boyce Thompson Institute, Cornell University, Ithaca, NY 14853, USA

## Abstract

Long noncoding RNAs (lncRNAs) are a large and diverse class of genes in eukaryotic genomes that contribute to a variety of regulatory processes. Functionally characterized lncRNAs play critical roles in plants, ranging from regulating flowering to controlling lateral root formation. However, findings from the past decade have revealed that thousands of lncRNAs are present in plant transcriptomes, and characterization has lagged far behind identification. In this setting, distinguishing function from noise is challenging. However, the plant community has been at the forefront of discovery in lncRNA biology, providing many functional and mechanistic insights that have increased our understanding of this gene class. In this review, we examine the key discoveries and insights made in plant lncRNA biology over the past two and a half decades. We describe how discoveries made in the pregenomics era have informed efforts to identify and functionally characterize lncRNAs in the subsequent decades. We provide an overview of the functional archetypes into which characterized plant lncRNAs fit and speculate on new avenues of research that may uncover yet more archetypes. Finally, this review discusses the challenges facing the field and some exciting new molecular and computational approaches that may help inform lncRNA comparative and functional analyses.

## Introduction

The basic definition of a long noncoding RNA (lncRNA) dictates that the RNA transcript must be 200 nucleotides or longer and not be translated into a protein. Traditional lncRNA definitions also exclude housekeeping RNAs, such as ribosomal (rRNA), transfer (tRNA), and small nuclear or nucleolar (sn/snoRNA; Amaral et al. 2011). This definition is problematic when considering RNAs that share similarity to portions of transposable or repetitive elements (Cho 2018) and those that give rise to small RNAs (sRNA) such as microRNAs (miRNAs), small interfering RNAs (siRNAs), and phasiRNAs (Wierzbicki et al. 2021). While the latter precursor RNAs can be considered bona fide lncRNAs, here we will not heavily focus on these RNAs, as there are many other excellent reviews written by experts on these RNA classes and pathways (Matzke and Mosher 2014; Wang et al. 2019a; Erdmann and Picard 2020; Liu et al. 2020). LncRNAs are also commonly defined based on the genomic context from which they are transcribed. For instance, lncRNAs are commonly separated into those that do not overlap other genes (intergenic lncRNAs or lincRNAs) and those that do. LncRNAs overlapping protein-coding genes are then separated based on the strand of overlap (antisense vs. sense) and context of overlap (intronic and exonic; Rinn and Chang 2012; Ma et al. 2013). Finally, a sometimes contentious point of the lncRNA definition concerns their noncoding nature. Current identification efforts rely on ORF length, protein similarity, and machine learning (ML) algorithms to distinguish between coding and noncoding RNAs. However, as discussed below, there are a number of described proteins and lncRNAs that defy these coding/noncoding definitions. Thus, determining whether a transcript is a lncRNA is a nontrivial task requiring both computational and molecular approaches, but one with important implications for plant biology.

In this review, we highlight the major contributions that plant researchers have made to lncRNA biology. We describe how discoveries around plant lncRNAs lit the path towards our functional understanding of these enigmatic transcripts, and how technological and algorithmic improvements have increased the number of identified plant lncRNAs from hundreds to thousands. We introduce the predominant computational algorithms and pipelines used to identify lncRNAs, and discuss where there are still challenges in lncRNA identification and analysis. We examine how plant lncRNAs fit into the functional paradigms developed for eukaryotic lncRNAs, with a particular focus on transcriptional regulation, as this is the predominant functional archetype seen to date for plant lncRNAs. We then discuss what is known about how lncRNAs themselves are regulated, and end with what we believe are the exciting new areas in lncRNA research in which we believe plants will continue to make major contributions.

## Historical introduction to lncRNAs

### LncRNAs in the pregenomics era

**Figure 1.**
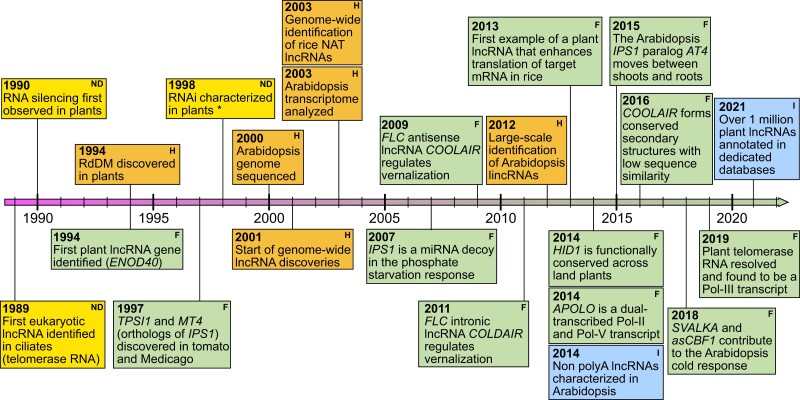
Important plant lncRNA discoveries over the past three decades. A timeline of seminal RNA and lncRNA discoveries with an emphasis on plant-specific pathways and mechanisms. *RNA interference and its characterization in plants were not covered in this review (Voinnet et al. 1998; Waterhouse et al. 1998). Green boxes with the letter ‘F’ in the top corner denote discoveries described in the plant lncRNA functional mechanisms section. Blue boxes with the letter ‘I’ in the top corner denote findings discussed in the section on major issues associated with lncRNA identification. Orange boxes with the letter ‘H’ in the top corner are discussed in the historical introduction, and yellow boxes with the letters ‘ND’ in the top corner are not explicitly discussed in the review, but place plant lncRNA discoveries in a greater context.

Plant biology has a rich history of supplying key discoveries in eukaryotic RNA biology (Fig. 1), including the initial observations of posttranscriptional gene silencing and stress-induced RNA-protein cytoplasmic aggregates, now referred to as stress granules (SGs; Nover et al. 1983; Matzke et al. 1989; Nover et al. 1989; Napoli et al. 1990). Plant-specific evolutionary innovations have also helped us understand how RNAs contribute to DNA methylation through RNA-dependent DNA methylation (RdDM; Wassenegger et al. 1994; Matzke and Mosher 2014). LncRNAs represent another class of RNA biology in which plant biologists have made seminal contributions to a field where foundational findings are generally attributed to nonplant model systems. Notably, many of these early lncRNAs were identified in agriculturally relevant species due to their involvement in physiologically important traits, highlighting the contributions even these nonmodel crop species have made to eukaryotic RNA biology.

Even prior to the sequencing of the Arabidopsis (*Arabidopsis thaliana*) genome in 2000 (Arabidopsis Genome Initiative 2000) and the beginning of the genomics era, a small number of lncRNAs were already emerging as functional players in a wide range of cellular activities. Due to the molecular and genetic technologies available, these first lncRNAs were identified based on their biological, rather than mechanistic, functions. By 2000, at least five lncRNAs had been functionally described: *ENOD40* (*EARLY NODULIN 40*) in *Medicago truncatula*, *CR20* (*CYTOKININ REPRESSED 20*) in cucumber (*Crocus sativus*), *GUT15* (*GENE WITH UNSTABLE TRANSCRIPT 15*) in tobacco (*Nicotiana tabacum*) and Arabidopsis, *MT4* in *M. truncatula*, and *TPSI1* (*TOMATO PHOSPHATE STARVATION INDUCED 1*) in tomato (*Solanum lycopersicum*). *ENOD40*, independently discovered in both *M. truncatula* and soybean (*Glycine max*), was the first lncRNA discovered in plants and plays a role in root nodulation in legumes (Crespi et al. 1994). Meanwhile, *MT4* and *TPSI1* are part of a dicot-conserved family of lncRNAs that respond to phosphate stress and, as we discuss below, contribute to appropriate responses to phosphate starvation (Burleigh and Harrison 1997; Burleigh and Harrison 1998; Burleigh and Harrison 1999; Liu et al. 1997; Bari et al. 2006; Franco-Zorrilla et al. 2007). *CR20* and *GUT15* represent a family of lncRNAs that are hormonally regulated, alternatively spliced, and conserved across angiosperms, yet there is limited functional data on these lncRNAs (Teramoto et al. 1996; MacIntosh et al. 2001; Plewka et al. 2018).

At the time of their discovery, it was unclear to the community if there was an RNA-specific molecular mechanism for these noncoding functional RNAs, as this ran counter to the predominant protein-centric views of molecular biology. However, many important observations were made to suggest a functional role for these early lncRNAs that was independent of any potential coding sequence. For instance, Crespi and co-authors determined that *ENOD40* likely performed its role as an RNA rather than a protein based on its free energy of folding being more similar to other noncoding RNAs compared to coding RNAs. And, despite transient overexpression of the soybean *ENOD40* resulting in the translation of a small signaling peptide, no peptide was observed from in vitro translation experiments or under native conditions in vivo (Crespi et al. 1994; van de Sande et al. 1996). These data, paired with the observation that *ENOD40* is most conserved outside its ORF, continue to support the model of *ENOD40* acting as a lncRNA. Thus, based on the definitions of noncoding RNAs of the era, these five genes represented puzzling, but exciting examples of functional lncRNAs.

Many experiments and observations from these early studies in plants laid the foundation for our current knowledge and definition of lncRNAs across all eukaryotes. While it was unclear to what degree these noncoding RNAs were present in plant transcriptomes, commonalities among them served as the basis for future identification efforts. For instance, these lncRNAs were typically expressed under very specific cellular or environmental conditions, a characteristic that holds true for many lncRNAs identified since. It is unclear if early researchers knew that lncRNAs, in general, were lowly expressed, as many of the early identified transcripts were likely the most abundant of the total lncRNA pool. At a molecular level, these early lncRNAs displayed mRNA-like attributes such as 5′ caps and poly-adenosine tails and were considered to be mRNA-like ncRNAs (Rymarquis et al. 2008). However, this is likely biased by how the original lncRNAs were identified and is not representative of all lncRNAs (discussed below). While the essential definition has not changed since these initial discoveries, we have more clarity on what it means to be a plant lncRNA, a more standardized definition, and much more functional data to guide mechanistic experiments.

### LncRNAs in the genomics era

In the decade that followed the sequencing of the Arabidopsis genome (Arabidopsis Genome Initiative 2000), there were numerous studies that noted widespread transcription in unannotated regions of plant genomes (Yamada et al. 2003; Meyers et al. 2004; Li et al. 2006; Chekanova et al. 2007; Li et al. 2007; Matsui et al. 2008; Okamoto et al. 2010). These observations aligned with reports from transcriptomic studies in animals, suggesting that pervasive transcription is a common feature in eukaryotic genomes (ENCODE Project Consortium et al. 2007; Kapranov et al. 2007). The first direct attempt at genome-wide plant lncRNA identification took place soon after the Arabidopsis genome sequence was published, when MacIntosh and co-authors identify 39 noncoding RNA candidates from two Arabidopsis expressed sequence tag (EST) collections representing ∼20,000 polyadenylated and size-selected transcripts (MacIntosh et al. 2001). Other analyses followed, primarily using ORF length (<100 amino acids (AA)) and similarity to known proteins, to identify a small number of mRNA-like noncoding RNAs in Arabidopsis and *M. truncatula* (Riaño-Pachón et al. 2005; Wen et al. 2007).

After these initial studies and during the following decade, our view of the lncRNA portion of the Arabidopsis transcriptome became clearer. Numerous groups, using varying scopes, technologies, and computational methodologies, identified suites of Arabidopsis lncRNAs (Marker et al. 2002; Osato et al. 2003; Wang et al. 2005; Hirsch et al. 2006; Wang et al. 2006; Amor et al. 2009; Song et al. 2009; Swiezewski et al. 2009). Most of these studies still relied on the ever-expanding databases of mRNA-focused ESTs and full-length cDNA sequences generated by a variety of groups and consortiums (Pontius et al. 2003; Castelli et al. 2004; Sakurai et al. 2005), however, their scope and methodology distinguished these studies. Some, such as Wang et al. (2005) and Wang et al. (2006), used full-length cDNA sequences from UniGene and RIKEN databases to identify ∼3,000 natural antisense transcripts (NATs). These NATs might share sequence complementarity in *cis* (directly overlapping a gene) or in *trans* (complementary sequences at separate loci). Other groups, such as Hirsch et al. (2006) and Amor et al. (2009), used a variety of strict criteria to identify high-confidence nonprotein-coding RNAs, including high GC content, other nucleotide biases, stable RNA structures, and features that would preclude successful translation. These findings uncovered a number of important functional lncRNAs. For example, the *APOLO* and *ALTERNATIVE SPLICING COMPETITOR* (*ASCO*) lncRNAs (discussed below) were both annotated as lncRNAs by Amor et al. (2009) prior to their biological function being described. In addition, algorithmic advances made it easier to annotate protein-coding genes accurately by incorporating comparative and transcriptomic information, e.g. MAKER (Holt and Yandell 2011), thereby making it easier to distinguish between unannotated protein-coding genes and lncRNAs. Thus, even before the large-scale adoption of RNA-sequencing in the 2010s, it was clear that thousands of transcriptionally active putative lncRNA loci existed in plant genomes.

### LncRNAs in the next generation RNA-sequencing era

As transcriptomic technologies improved in the 2010s (e.g. higher density tiling arrays and next generation RNA-sequencing), it became cheaper and easier to perform more comprehensive lncRNA identification efforts that spanned numerous tissues or conditions. One of the first of these studies came from Liu et al. (2012) in which they used 200 publicly available and custom-made tiling arrays, as well as their own RNA-seq data from four tissues to identify nearly 7,000 lincRNAs and a similar number of NAT-lncRNAs. These public arrays targeted poly-A RNA from 14 Arabidopsis mutants, 18 heat treatments, and 6 different plant tissues. Importantly, reproducibility across multiple tiling arrays was used as a criterion to generate high-confidence lncRNAs. Finally, Liu et al.’s study used both tiling array technology and RNA-seq, allowing for a near-direct comparison of technologies for lncRNA discovery. While ∼40% of their 6,480 lincRNAs had some measure of RNA-seq-based transcriptional support, fewer than 300 of them were fully supported by their relatively deep sequencing (∼250 million reads/tissue). This updated annotation was instrumental in the discovery of many now functionally characterized lncRNAs, such as *lncCOBRA1*, *FLORE*, *DRIR*, and *AGAMOUS (AG)-incRNA4* (Henriques et al. 2017; Qin et al. 2017; Wu et al. 2018; Kramer et al. 2022). In addition, these data highlighted differences between tiling arrays and RNA-seq, and also pointed to the necessity for sequencing breadth being just as, or more, important as depth when attempting to comprehensively identify lncRNAs.

A number of other lncRNA identification efforts closely followed the initial work by Liu et al. (2012). Importantly, these included further efforts in Arabidopsis and many agriculturally important species (Boerner and McGinnis 2012; Moghe et al. 2013; Li et al. 2014a; Zhang et al. 2014; Shumayla et al. 2017). Similar findings to those in Arabidopsis were found in maize (*Zea mays*) by Boerner and McGinnis where they identified ∼2,500 lncRNAs using a public dataset of full-length cDNA sequences (Boerner and McGinnis 2012). About half of these novel lncRNAs were categorized as siRNA precursors. This finding differs from Arabidopsis studies, where Liu et al. found that ∼2.5% of their identified lincRNAs were sRNA associated. In support of Boerner and McGinnis’ study, a follow-up lncRNA identification study in Maize using ESTs and RNA-seq from diverse tissues found evidence for over 20,000 lncRNAs of which more than 90% were sRNA precursors (Li et al. 2014b). In the past decade since Liu et al. RNA-seq-based lncRNA identification efforts have expanded to almost every model or agronomically relevant plant species, with remarkably consistent findings, lncRNAs are abundant but lowly expressed, making their identification difficult but worth the effort.

## Predominant methods of identifying lncRNAs in plants

The flood of transcriptional evidence supplied by next generation RNA-sequencing data necessitated improvements in how lncRNAs are identified from these data. Traditionally, lncRNA identification pipelines discard transcripts based on size (<200 nts), abundance (varies, e.g., less than 1 transcript per million), similarity to known genes (e.g., using Rfam (an RNA family database, Griffiths-Jones et al. 2003) and Pfam (The protein familes database, Mistry et al. 2021), and ORF length (>100 AA). While imperfect, this approach helped to identify a number of functionally important lncRNAs in eukaryotes (Cabili et al. 2011; Liu et al. 2012). Over time, additional approaches have been developed that build on characteristics of previously identified lncRNAs to better determine what is coding and noncoding. In this section, we summarize some of the computational resources, algorithms, and strategies that have been developed over the past decade to aid in lncRNA annotation and functional prediction.

### Algorithms

To date, there are more than 30 distinct algorithms and pipelines developed for lncRNA identification. In general, many of these algorithms (Table 1) rely on sequence intrinsic features, such as ORF length and composition, coding potential, and sequence decomposition (i.e. k-mers). Additionally, some of these bioinformatic packages require reference genome and annotation files (referred to as *alignment-based*), whereas others do not (*alignment-free*; Table 1). Alignment-based algorithms such as CPC2 and PlncPRO tend to be faster and more accurate, as the input transcripts typically lack sequencing errors and have a more accurate gene structure than those used in alignment-free methods (i.e. de novo assembly; Kang et al. 2017; Singh et al. 2017). However, in nonmodel systems where genomes or genome annotations are lacking, alignment-free approaches such as PLEK (predictor of long non-coding RNAs and messenger RNAs based on an improved k-mer scheme) and CNCI (Coding-Non-Coding Index) are quite useful (Sun et al. 2013; Li et al. 2014a; Schneider et al. 2017; Guo et al. 2019). Of note: for all algorithms, the input information is a set of transcript sequences—thus alignments are not necessary, but alignment-free methods have been explicitly designed to overlook sequencing or transcript assembly errors that might influence feature comparisons.

**Table 1.** Algorithms and pipelines used to identify lncRNAs from RNA-sequencing data

Name	Method (specific algorithm)	Features for classification	Organism	Manner	References
Annocript	Genomic feature-based	Homology, ORF lengths, other	Animals	Alignment-based	Musacchia et al. (2015)
BASiNET	DL (decision tree on complex network)	Topological measures of sequence networks	Vertebrates	Alignment-free	Ito et al. (2018)
CNCI	ML (support vector machine (SVM))	Adjoining nucleotide triplets, ORF structure, other	Vertebrates	Alignment-free	Sun et al. (2013)
CNIT	ML (XGBoost)	66 features, including most-like CDS, adjoining nucleotide triplets, etc.	Animals and plants	Alignment-based	Guo et al. (2019)
COME	ML (random forest)	Nine features, including sequence-derived, expression, and histone features	Human	Alignment-based	Hu et al. (2007)
CPAT	Logistic regression	ORF size and coverage, Fickett score, hexamer usage	Animals	Alignment-free	Wang et al. (2013)
CPC2	ML (random forest)	ORF length, integrity, Fickett score, isoelectric point	Animals	Alignment-free	Kang et al. (2017)
CREMA	ML (multiple)	mRNA length, ORF length, GC content, conservation, other	Animals and plants	Alignment-based	Simopoulos et al. (2019)
DeepLnc	DL (deep neural networks)	K-mer	Human	Alignment-free	Tripathi et al. (2016)
Evolinc	Pipeline	Utilizes CPC2 and common lncRNA heuristics	Animals and plants	Alignment-based	Nelson et al. (2017)
FEElnc	ML (random forest)	Relation to known transcripts, ORF characteristics, other	Mammals	Alignment-free	Wucher et al. (2017)
iSeeRNA	ML (SVM)	Conservation, ORF length, ORF proportion	Animals	Alignment-based	Sun et al. (2013)
LGC	ML	ORF length and GC content	Animals and plants	Alignment-free	Wang et al. (2019a)
LncADeep	DL (deep belief network)	Sequences intrinsic and homology feature in a deep belief network	Human	Alignment-free	Yang et al. (2018)
LncFinder	ML (multiple)	ORF, structure, physiochemical property, other	Animals and plants	Alignment-free	Han et al. (2019)
lncRNA-ID	ML (random forest)	Ribosome interaction, protein conservation features, other	Animals	Alignment-based	Achawanantakun et al. (2015)
lncRNA-MFDL	DL (deep stacking networks)	ORF, k-mers, structure, other	Human	Alignment-free	Fan and Zhang (2015)
lncRNA-screen	Genomic feature-based	Relation to known transcripts, no small RNA overlap, other	Animals	Alignment-based	Gong et al. (2017)
lncRNAnet	DL (recurrent neural network)	Intrinsic features extracted by recurrent neural networks	Human	Alignment-free	Baek et al. (2018)
lncRScan-SVM	ML (SVM)	Transcript length, stop codon presence, conservation, other	Animals	Alignment-based	Sun et al. (2015)
LncScore	Logistic regression	Coding potential, ORF characteristics, exon hexamers, and GC content	Animals	Alignment-free	Zhao et al. (2016)
longdist	ML (SVM)	Nucleotide pattern frequencies, ORF characteristics	Animals	Alignment-based	Schneider et al. (2017)
PLEK	ML (SVM)	K-mer	Vertebrates	Alignment-free	Li et al. (2014a)
PLIT	ML (iterative random forests)	ORF characteristics, codon-bias, other	Plants	Alignment-free	Deshpande et al. (2019)
PLncPRO	ML (random forest)	Homology to known proteins, codon-bias, length, other	Plants	Alignment-based	Singh et al. (2017)
PlncRNA-HDeep	DL	Sequence composition encoded into vectors	Plants	Alignment-free	Meng et al. (2021)
PORTRAIT	ML (SVM)	Nucleotide composition, translated ORF characteristics, other	Fungi	Alignment-based	Arrial et al. (2009)
RNAplonc	ML (multiple)	16 features including K-mer, sequence length, GC content, coding potential	Plants	Alignment-free	Da Negri et al. (2019)

Abbreviations: BASiNET, BiologicAl Sequences NETwork; CDS, coding sequence; CNIT, Coding-Non-Coding Identifying Tool; COME, a coding potential calculation tool based on multiple features; CPAT, Coding-Potential Assessment Tool; CREMA, Classifying RNA by Ensemble Machine learning Algorithm; DL, Deep learning; FEElnc, FlExible Extraction of LncRNAs; lncRNA-ID, Long non-coding RNA IDentification; LGC, ORF Length and GC content.

The algorithms incorporated into many researcher's lncRNA identification workflows, such as the commonly used CPC2 (coding potential calculator 2) (Kang et al. 2017), were developed and trained predominantly on vertebrate lncRNAs. Given lineage-specific genomic differences (e.g. GC content), models trained on vertebrate lncRNAs may incorrectly assign plant lncRNAs. Recognizing this potential issue, a few tools (Table 1) have been developed and optimized specifically for plant lncRNA identification, including PlncRNA-HDeep (Plant LncRNA hybrid deep learning model) and RNAplonc, which both employ deep learning approaches (Da Negri et al. 2019; Meng et al. 2021), as well as PlncPRO (Plant Long Non-Coding Rna Prediction by Random Forests) and PLIT (Plant LncRNA Identification Tool) which utilize random forest models (Singh et al. 2017; Deshpande et al. 2019). While each of these algorithms was more accurate in predicting plant lncRNAs, it is likely how the models were trained (on plant lncRNAs), rather than the machine learning approach itself that lends them this higher accuracy. A comparison of each of these tools using the same well-curated set of plant lncRNAs would be useful in determining which was the most appropriate for a given set of input transcripts.

### Integrative pipelines

In efforts to streamline lncRNA discovery and evolutionary analysis across large datasets, pipelines, such as Evolinc (Nelson et al. 2017), have been developed. Evolinc utilizes multiple out of the box ML algorithms (e.g. CPC2) as well as traditional heuristics (e.g. transcript length and similarity to known proteins) for lncRNA identification and is simple to use, but generally is not amenable to changes in the underlying filtering mechanisms and reliant on predefined lncRNA features. Another useful aspect of Evolinc is the evolutionary portion of the pipeline, which searches for sequence homologs in a user-defined set of related species (Nelson Dittrich and Nelson 2022). Another useful pipeline is lncRNA-screen (Gong et al. 2017), which is designed to incorporate additional genomic features, such as histone marks, HiC data, and transcript abundance, to better functionally annotate already identified lncRNAs. Similarly, LncADeep is a deep learning approach to identify and functionally annotate lncRNAs, and furthermore, infer putative protein interaction partners based on numerous sequence characteristics (Yang et al. 2018). As the community settles on a few primary lncRNA identification algorithms, these simplified pipelines will likely become automated aspects of genome annotation projects.

The increasing number of novel algorithms and computational pipelines for annotating lncRNAs has invited challenging discourse around “best” identification practices. Some pressing questions at the moment are: How do users determine which method is best suited to their data and how does the community compare lncRNAs identified using different methods? One potential solution would be for each research group annotating a novel lncRNA, or group of RNAs, to utilize multiple approaches and take the intersection of those approaches (i.e. predictions shared between those approaches). However, selecting seemingly disparate algorithms that utilize different lncRNA features for identification raises additional concerns about which is most appropriate. In addition, there are potentially species-specific tradeoffs to consider, particularly when annotating lncRNAs in nonmodel plant systems lacking reference genomes. Possible solutions would be for the plant community to (1) develop a common test dataset for benchmarking purposes and (2) have multiple groups collaborate to perform this benchmarking on extant algorithms to compare performance and accuracy.

## Major issues associated with lncRNA identification in the genomics era

The many attempts at annotating lncRNAs in plant transcriptomes have revealed a number of key properties that have made their identification problematic. One of these issues pertains to sequencing resolution and appropriate supporting data at cellular, tissue, and organismal scales. For example, even though most model plant species have sufficient transcriptomic data across all major plant organs and developmental stages, very few concerted efforts have been undertaken to utilize these data to annotate lncRNAs. In addition, companion data that would provide greater support for lncRNA annotation, such as information about transcription start sites (cap analysis of gene expression (CAGE); Kawaji et al. 2014), structure and protein interaction (protein interaction profiling sequencing; Silverman et al. 2014), and degradation (e.g., parallel analysis of RNA ends or genome-wide mapping of uncapped and cleaved transcripts; Gregory et al. 2008; German et al. 2009; Willmann et al. 2014), are often limiting. This restricts the confident development of lncRNA repertoires for many species to only a few commonly sampled tissues, often leaves or seedlings. Because of the importance of comparative approaches to characterize lncRNAs, expanding the tissues and environmental conditions used to identify and profile their expression will help to propel their functional characterization. This should be a priority for plant lncRNA biology as much of these data already exist and are publicly available.

### Sampling bias

LncRNAs have classically been thought of as mRNA-like transcripts. As a result, most identification efforts have sampled polyadenylated (polyA) pools of RNA. However, polyA-focused studies may overlook a significant fraction of the ncRNA transcriptome and thus ignore biologically significant lncRNAs. In animals, there are thousands of non-polyA noncoding transcripts which do not fall into the classical definition of housekeeping RNAs (rRNA, tRNA, sn/snoRNA; Livyatan et al. 2013). The initial characterization efforts of non-polyA plant ncRNAs came in 2013 and 2014 (Liu et al. 2013; Di et al. 2014; Wang et al. 2014b), with Di and co-authors focusing specifically on non-polyA lncRNAs in Arabidopsis. Di et al. used rRNA depletion and polyA minus RNA-seq in four stresses to identify lncRNAs lacking polyA tails. More recently, a combined transcriptomic approach that sampled both polyA and non-polyA lncRNAs and their localization within the cell uncovered a suite of stress-responsive lncRNAs, including *MAS* (discussed below; Zhao et al. 2018). The biological significance of these non-polyA and likely nonpolymerase II (non-Pol-II) transcribed ncRNAs is hinted at by the discovery of the highly conserved lncRNA telomerase RNA (*TR*), a Pol-III transcript whose discovery relied on surveying ribosomal depleted RNA-seq libraries (Fajkus et al. 2019; Song et al. 2019; Dew-Budd et al. 2020). Separately, some of these non-polyA transcripts may be associated with or are produced by the RdDM pathway and therefore have implications in epigenetic silencing (Tsuzuki et al. 2020). Indeed, the RdDM pathway, and plant sRNA pathways in general, may be substantial contributors to the overall lncRNA pool in plants. Thus, we believe that it may be necessary to shift RNA-sequencing efforts to ribo-depleted libraries in order to capture the noncoding RNA portions of plant transcriptomes in a more complete manner.

### Low expression

Perhaps the greatest difficulty in annotating lncRNAs comes from their intrinsic characteristics. Unannotated transcripts that are fed into lncRNA identification pipelines are typically mono-exonic transcripts with low expression, and may have insufficient data to infer gene structure. Determining which of these lowly expressed, mono-exonic transcripts fit into the definition of a lncRNA is further complicated by the relatively broad, and nonstandardized, definition of a lncRNA. For example, a typical deep RNA-sequencing experiment identifies thousands of unannotated and lowly expressed transcripts which fit the criteria of a lncRNA (Liao et al. 2017; Wang et al. 2020). However, many of these transcripts have inconsistent expression patterns and may not appear in independent RNA-sequencing data from the same tissues (Palos et al. 2022). In addition, their low expression makes it difficult to distinguish them algorithmically from transcriptional noise derived from deep sequencing. It is important to note here that low expression does not equate to lack of function, as a lncRNA may have rapid turnover or be functional at very low stoichiometries (Unfried and Ulitsky 2022). Emerging work in human cells suggests that many lncRNAs may function by initiating liquid–liquid phase separation, a phenomenon whereby distinct membrane-less compartments form within the cell and contribute to cellular stability. These compartments would also explain the substoichiometric nature of lncRNA copy-number and function within the cell (Guo et al. 2021; Wu et al. 2021). To navigate issues assembling lowly expressed transcripts, some groups have suggested filtering out single exon transcripts, as well as those that are within 500 base pairs of another gene (Cabili et al. 2011; Cemel et al. 2017). These are rational and conservative decisions but do not work well with smaller genomes, such as Arabidopsis, and fail to consider the large number of functionally described mono-exonic lncRNAs in plants (Franco-Zorrilla et al. 2007; Wang et al. 2014a; Fajkus et al. 2019). In short, there always seem to be exceptions to the most carefully thought-out lncRNA definition. Thus, until high-throughput genetic screens such as Perturb-seq (Adamson et al. 2016) are translated to plant systems, we would argue that replication and variability in expression in response to stimuli are key. To sum up, a high-confidence lncRNA must be identified in multiple experimental and biological replicates, and the expression of the lncRNA should be induced under specific circumstances. As mentioned below, the induction pattern can assist in functional prediction.

### Poor sequence conservation

Beyond Arabidopsis and other model plants, the increased number of sequenced genomes and transcriptomes largely enabled the comparative and evolutionary studies of plant lncRNAs (Mohammadin et al. 2015; Nelson et al. 2016; Simopoulos et al. 2019; Corona-Gomez et al. 2020; Fesenko et al. 2021; Zhu et al. 2022). These comparative analyses suggest that plant lncRNAs are more evolutionarily labile, with much shorter apparent evolutionary halflives than those seen in vertebrates (Cabili et al. 2011; Necsulea et al. 2014; Washietl et al. 2014; Hezroni et al. 2015). Sequence homologs are difficult to find in even closely related species for most plant lncRNAs. In addition, homology does not necessarily coincide with collinearity (synteny). Interestingly, sequence-divergent lncRNAs, transcribed adjacent to orthologous protein-coding genes, have been observed in the mustards, suggesting that transcriptional conservation may be more important than sequence for certain lncRNAs (Walden et al. 2020; Palos et al. 2022). For the subset of lncRNAs with identifiable homologs, conserved domains, structures, splice sites, and interaction partners have been discerned. Interestingly, even these conserved lncRNAs are rarely identified through sequence-based homology searches outside of a plant family, requiring more detailed co-variation and structure-based models for homology inference (Hawkes et al. 2016; Fajkus et al. 2019, 2021). Thus, plant lncRNAs may be divided into at least three evolutionary classes: (1) the species-specific lncRNAs that appear to make up the majority of lncRNA populations, (2) the lncRNAs with potential transcriptional and positional conservation, but little sequence conservation, and (3) the much smaller number of lncRNAs with easily observable modes of conservation more typical of protein-coding genes. These three evolutionary classes are likely linked to functional mechanism and biological significance, and will help guide future exploration into plant lncRNAs. Thus, developing high-throughput comparative tools to evolutionarily classify lncRNAs will be critical for future functional work.

### Lack of a common lncRNA definition

Difficulties in coalescing around a common definition for lncRNAs are even visible in the repositories meant to serve the plant lncRNA community. There are three comprehensive and plant-specific lncRNA databases, with over one million lncRNAs across close to 100 species, that have been developed in the last 5 yr (Szcześniak et al. 2019; Jin et al. 2021a; Di Marsico et al. 2022). GreeNC (http://greenc.sequentiabiotech.com/wiki2/; Di Marsico et al. 2002) is unique among these databases in that it utilized a purely in silico approach to identify lncRNAs from previously annotated transcripts. They identified ∼500,000 putative lncRNAs from 94 plant and algae species. A unique and helpful resource that GreeNC provides is their description of lncRNA orthogroups which facilitates accessible comparative analyses and prediction for function (Di Marsico et al. 2022). A notable feature missing from GreeNC is the information pertaining to lncRNA expression. In contrast, both CANTATAdb (http://cantata.amu.edu.pl/; Szcześniak et al. 2019) and PLncDB (www.tobaccodb.org/plncdb/; Jin et al. 2021a) have curated publicly available expression data to predict lncRNAs. To date, over 1.2 million lncRNAs across 80 plant species and over 200,000 lncRNAs across nearly 40 plant species are represented in PLncDB and CANTATAdb, respectively. The data curated by PLncDB are especially relevant for hypothesis generation, as it includes experimental, expression, and intermolecular regulatory network information. While the developers of PLncDB annotated an expansive repertoire of lncRNAs utilizing diverse sets of sequencing data, those behind CANTATAdb arguably took a more conservative approach in data utilization and lncRNA identification. LncRNAs in the GreeNC and PLncDB databases were defined with an ORF cutoff of 120 AA, whereas those in CANTATAdb were defined by an ORF cutoff of 100 AA. Additionally, only paired-end sequencing data were used to identify lncRNAs for the CANTATAdb, which improves read mapping quality and transcript assembly. While the utility of these resources is apparent, the lack of harmonization across them, as well as the different ways in which they define lncRNAs, makes it difficult to easily compare across them. Both to facilitate discovery and reduce upkeep costs, we would propose a unified database that was linked to species-specific genomic resources (e.g. the Arabidopsis information resource or MaizeGDB; Swarbreck et al. 2008; Portwood et al. 2019).

### Plant lncRNA functional mechanisms

**Figure 2.**
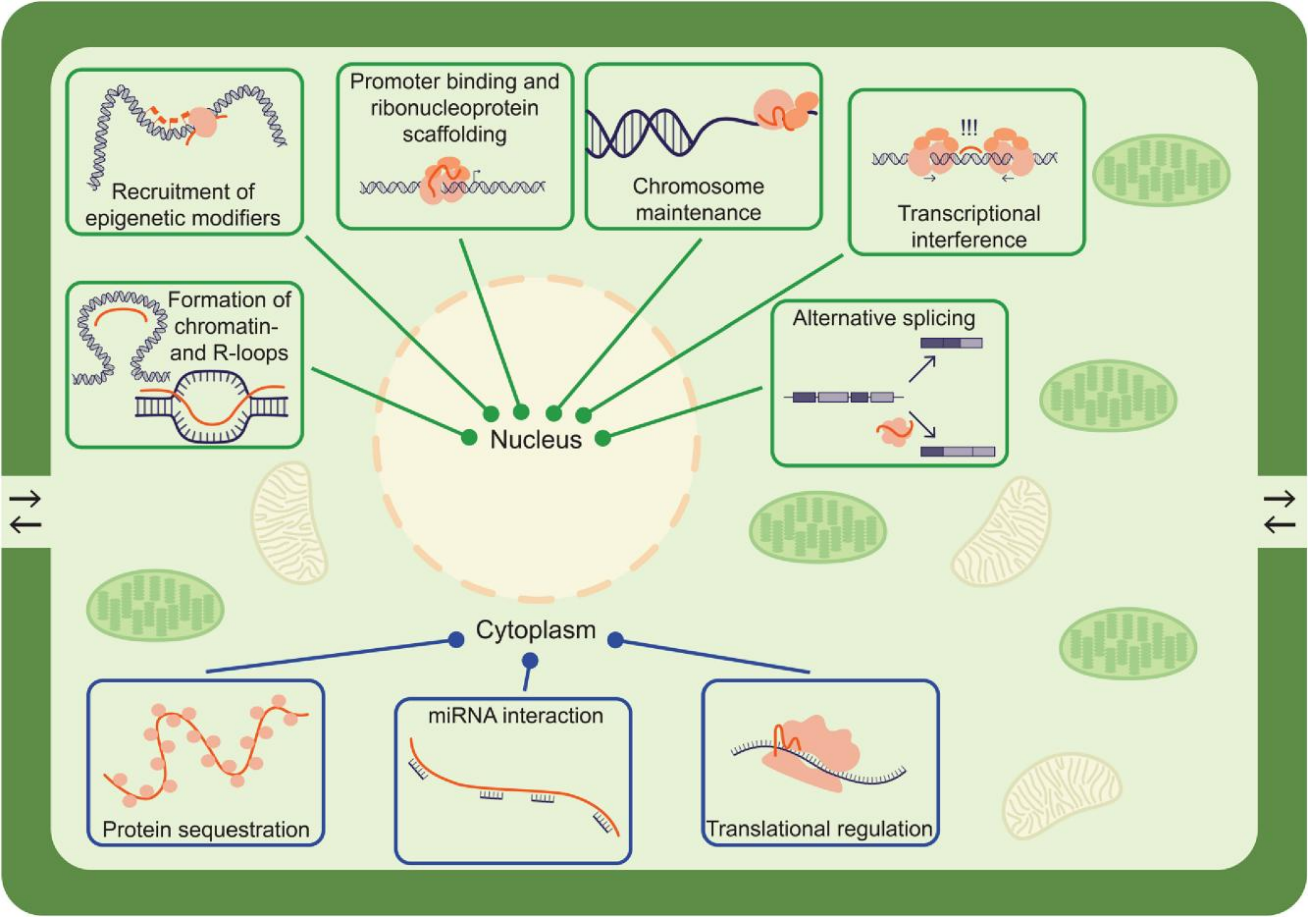
Functional lncRNA archetypes. A simplified cellular diagram displaying various nuclear or cytoplasmic mechanisms reported in plants. LncRNAs are shown as thick orange lines in each diagram. Nuclear activities are shown in green boxes with lines pointing towards the nucleus, whereas cytoplasmic activities are shown in blue boxes with lines pointing toward ‘Cytoplasm’.

Many of the functionally characterized plant lncRNAs are predominantly nuclear-localized and exhibit some role in transcriptional or posttranscriptional gene regulation (Fig. 2**)**. For the sake of clarity, we have separated these mechanisms below, with an additional section for lncRNAs whose function falls outside of regulating gene expression. Each of the described lncRNAs, their functional archetype, and when and where they were identified, can be found in Table 2.

**Table 2.** Functionally and mechanistically annotated plant lncRNAs

lncRNA name	Subcellular localization	Species of initial identification	Initial publication date	Functional archetype (Fig. 2)
*AG-incRNA4*	N	Arabidopsis	Wu et al. (2018)	Recruitment of epigenetic modifiers
*APOLO*	N	Arabidopsis	Ariel et al. (2014)	Formation of chromatin- and r-loops + recruitment of epigenetic modifiers
*ASCO*	N	Arabidopsis	Bardou et al. (2014)	Alternative splicing
*COLDAIR*	N	Arabidopsis	Heo and Sung (2011)	Recruitment of epigenetic modifiers
*COLDWRAP*	N	Arabidopsis	Kim and Sung (2017)	Formation of chromatin loop + recruitment of epigenetic modifiers
*COOLAIR*	N	Arabidopsis	Liu et al. (2010)	Recruitment of epigenetic modifiers
*DRIR*	N	Arabidopsis	Qin et al. (2017)	ND
*Ef-cd*	N	Rice	Fang et al. (2019)	Recruitment of epigenetic modifiers
*ELENA1*	N	Arabidopsis	Seo et al. (2017)	Promoter binding and ribonucleoprotein scaffolding
*ENOD40*	N, C	Medicago	Crespi et al. (1994)	Alternative splicing + protein sequestration
*FLORE*	N	Arabidopsis	Henriques et al. (2017)	ND
*FLAIL*	N	Arabidopsis	Jin et al. (2021b)	ND
*HID1*	N	Arabidopsis	Wang et al. (2014b)	Promoter binding and ribonucleoprotein scaffolding
*IPS1*	C	Tomato and Medicago	Burleigh and Harrison (1997) and Liu et al. (1997)	miRNA interaction
*LAIR*	N	Rice	Wang et al. (2018)	Recruitment of epigenetic modifiers
*lncCOBRA1*	N	Arabidopsis	Kramer et al. (2022)	Ribonucleoprotein scaffolding
*lncRNA39896*	C	Tomato	Hong et al. (2022)	miRNA interaction
*MARS*	N	Arabidopsis	Roulé et al. (2022)	Formation of chromatin loop + recruitment of epigenetic modifiers
*MAS*	N	Arabidopsis	Zhao et al. (2018)	Recruitment of epigenetic modifiers
*MISSEN*	C	Rice	Zhou et al. (2021)	Protein sequestration
*PHO1;2-NAT*	C	Rice	Jabnoune et al. (2013)	Translational regulation
*SEP3 circRNA*	N	Arabidopsis	Conn et al. (2017)	Formation of R-loop + alternative splicing
*SVALKA/asCBF1*	N	Arabidopsis	Kindgren et al. (2018)	Transcriptional interference
*TR*	N	Onion and other land plants	Fajkus et al. (2019) and Song et al. (2019)	Chromosome maintenance
*TWISTED LEAF*	N	Rice	Liu et al. (2018)	Recruitment of epigenetic modifiers

## Pretranscriptional regulation of gene expression by lncRNAs

### Modulating expression through the formation of chromatin loops

LncRNAs can mediate changes in gene expression through alterations to chromatin topology. One prominent and well-studied example of this functional class is the lncRNA *APOLO* (*AUXIN REGULATED PROMOTER LOOP*) and its role in regulating the expression patterns of auxin-responsive genes (Ariel et al. 2014, 2020; Mas and Huarte 2020; Moison et al. 2021). Upon auxin treatment, the *APOLO* locus undergoes chromatin relaxation. This results in RNA Pol-II expression of both *APOLO* and upstream neighbor, *PINOID* (*PID*), a protein kinase associated with polar auxin transport (Friml et al. 2004). In a self-regulating cycle, Pol-II *APOLO* accumulation encourages the recruitment of RNA polymerase V (Pol-V) and the production of Pol-V *APOLO* variants. Accumulation of the Pol-II *APOLO* variant leads to recruitment of the polycomb repressive complex 1 and 2 (PRC1/2) which maintain and deposit the repressive H3K27me3 (histone3, lysine 27 trimethylation) mark, respectively. This subsequently leads to repression at the locus (partially through the RdDM pathway), a return of the repressive chromatin loop, and loss of *PID* expression. Interestingly, *APOLO* is also able to navigate to target sites *in trans* via sequence complementarity, without a requirement for topological association (Ariel et al. 2020). To our knowledge, this appears to be one of the first examples of a eukaryotic lncRNA impacting chromatin topology in *cis* and in *trans* merely through sequence complementarity. In sum, dual transcription of the *APOLO* locus facilitates tight transcriptional control of auxin-responsive genes in a highly dynamic fashion (Ariel et al. 2020).

A new and compelling avenue that lncRNAs have been shown to act in is the regulation of biosynthetic gene clusters. In the last two decades, numerous examples of genomic co-localized biosynthetic gene clusters have been identified and are often co-regulated (Bharadwaj et al. 2021; Polturak and Osbourn 2021). LncRNAs derived from biosynthetic gene clusters present a unique mechanism for the cis-regulation of gene clusters and associated genetic elements. In a mechanism similar to *APOLO*, *MARS* (*MARneral Silencing*), a lncRNA transcribed from within the Arabidopsis marneral cluster, also functions as a chromatin topology modulator (Roulé et al. 2022). In response to the hormone abscisic acid (ABA), elevated *MARS* expression likely decoys PRC1 away from the marneral cluster and facilitates chromatin loop formation; this seems to enhance the expression of the *MARNERAL SYNTHASE1* gene. This chromatin loop brings the marneral cluster closer to distal ABA-responsive elements, effectively modulating the expression of the marneral gene cluster during seed germination and osmotic stress (Roulé et al. 2022).

In plants, as in many organisms, developmental transitions need to be connected to environmental cues, and thus are often under tight and tunable regulation. In Arabidopsis and throughout the Brassicaceae, the *FLOWERING LOCUS C* (*FLC*) gene is a critical regulator of vernalization, the process that initiates flowering after prolonged cold exposure (Whittaker and Dean 2017). *FLC* is a MADS-box transcription factor that represses a suite of floral activation genes. Therefore, *FLC* expression is repressed upon cold exposure to allow for the appropriate timing of flowering to facilitate successful reproduction. Nested within the *FLC* locus is a group of lncRNAs that play critical roles in the vernalization response through their repression of *FLC*. These lncRNAs have been reviewed extensively elsewhere (Wang and Chekanova 2017; Whittaker and Dean 2017; Lucero et al. 2021), so we will only touch on them here in the context of how they relate to archetypal lncRNA functions. *COLDWRAP* is a lncRNA transcribed from the repressed promoter of *FLC* and contributes to the establishment of H3K27me3 at the *FLC* promoter through interactions with PRC2. In addition, *COLDWRAP* is necessary to form a repressive intragenic chromatin loop at the 5′ end of the *FLC* gene; this loop appears necessary for spreading of H3K27me3 (Kim and Sung 2017). This PRC2-*COLDWRAP* interaction plays a part in preventing precocious flowering. As we discuss below, the regulation of *FLC* is complex and provides examples of multiple lncRNA archetypes.

The mechanisms of *APOLO*, *MARS*, and *COLDWRAP* collectively represent a common archetype of regulating the formation and regulation of chromatin loops, paired with interactions with repressive histone complexes (Fig. 2). As more plant lncRNAs are characterized over the next decade, we anticipate a large number of lncRNAs to fall into this functional archetype.

### Modulating expression in a chromatin loop-independent manner

There are an accumulating number of plant lncRNAs that can regulate gene expression by recruiting histone modifiers independent of any reported chromatin looping. Along with *COLDWRAP* as mentioned above, the lncRNAs *COOLAIR* and *Antisense Long* (*ASL*) are both necessary for the proper transcriptional repression of *FLC*. *COOLAIR* is a capped, polyadenylated, and alternatively spliced lncRNA that is transcribed from the antisense strand of *FLC*, initiating near the terminator of *FLC*. *COOLAIR* is a component of the vernalization-controlled flowering pathway, in that it is upregulated during cold conditions and contributes to the initial repression of *FLC* by promoting a repressed chromatin state through removal of active epigenetic marks, namely H3K36me3 and H3K4me1 (Liu et al. 2010; Csorba et al. 2014; Tian et al. 2019; Fang et al. 2020). This removal of epigenetic marks seems to occur through the H3K4 demethylase, FLOWERING LOCUS D (Fang et al. 2020). *ASL* is transcribed from the same promoter as COOLAIR, is also alternatively spliced, but is not polyadenylated (Shin and Chekanova 2014). While *COOLAIR* and *COLDWRAP* are expressed in response to cold, *ASL* biogenesis is dependent upon components of the exosome and thereby likely regulates *FLC* in a temperature-independent (autonomous) manner. *ASL* RNA immunoprecipitation experiments and epigenetic analyses revealed that *ASL* physically associates with both exosomal proteins and H3K27me3 regions within the *FLC* locus (Shin and Chekanova 2014). Thus, in the characterization of *ASL*, Shin and Chekanova established a mechanism for how FLC could be regulated by both the vernalization and autonomous flowering pathways in Arabidopsis.

Another repressive regulatory lncRNA arising from the *FLC* locus is *COLDAIR*, transcribed from the sense strand of the first intron of *FLC*. Working in tandem with *COOLAIR* to epigenetically silence *FLC*, *COLDAIR* associates with the *FLC* locus, recruiting the PRC2 complex to deposit H3K27me3 (Kim and Sung 2017). H3K27me3 further reinforces transcriptional repression of the *FLC* locus during vernalization. In addition to the *FLC*-derived lncRNAs, *MAS* (antisense to the *MADS AFFECTING FLOWERING4*-*MAF4*) is also essential to maintaining proper flowering control in Arabidopsis (Zhao et al. 2018). *MAF4* encodes for another MADS-box transcription factor that is paralogous to *FLC* and also acts as a vernalization-regulated floral repressor (Gu et al. 2013). During prolonged cold temperatures, *MAS* and *MAF4* expression are consistently low until a burst of expression at 20 d post cold exposure when expression peaks for both transcripts. This burst of *MAF4* expression is hypothesized to prevent premature flowering during the early vernalization response (Kim and Sung 2013). *MAS* was shown to directly influence the expression of *MAF4* through recruitment of the COMPASS-like complex to the sense strand of *MAF4* which deposits H3K4me4 to promote the transcriptional burst of *MAF4* (Zhao et al. 2018). The transcription of *MAF4* enhances the suppression of early flowering (Zhao et al. 2018). In addition to governing floral transitions and morphogenesis, lncRNAs have the capacity to epigenetically drive tissue-specific expression patterns. In Arabidopsis, an intronic lncRNA of *AG*, *AG-incRNA4* recruits PRC2 components to facilitate *AG* repression. *AG*, encoding for yet another MADS-box transcription factor, is a key regulator of stamen and carpel identity in Arabidopsis flowers that must be repressed in vegetative tissues (Pelayo et al. 2021). *AG-incRNA4* interacts with PRC2 to promote the deposition of H3K27me3 at *AG* in vegetative tissues, in a mechanism remarkably similar to *COLDAIR*-mediated repression of *FLC* (Wu et al. 2018). Both *AG-incRNA4* and *COLDAIR* directly bind the PRC2 component CURLY LEAF to recruit the complex to DNA (Wu et al. 2018; Tian et al. 2019). Thus, cis-regulation of MADS-box transcription factors through overlapping lncRNAs might be a widespread mechanism to ensure specificity of expression in this family of transcription factors.

In rice, *LAIR* (*LRK antisense intergenic RNA*), a nuclear-localized lncRNA found within the *leucine-rich kinase* (*LRK*) gene cluster functions as a positive regulator of the *LRK* cluster in a PRC2-dependent manner. *LAIR* directly interacts with the histone modifiers OsMOF and OsWDR5, components of an H4K16 acetyltransferase and associated protein complex (Taipale et al. 2005; Yang et al. 2014; Wang et al. 2018). This interaction positively regulates the expression of *LRK* genes through the deposition of H4K16ac and H3K4me3, and overexpression of *LAIR* ultimately leads to an increase in grain yield (Wang et al. 2018). *Ef-cd* (*early flowering-completely dominant*), an antisense lncRNA transcribed from the *OsSOC1* locus, represents another example by which yield is manipulated by lncRNAs. *OsSOC1* is the putative ortholog of the Arabidopsis *SUPPRESSOR OF OVEREXPRESSION OF CO1*, a floral integrator that is regulated by FLC and other floral regulators (Lee and Lee 2010). Although it is mechanistically unclear how *Ef-cd* functions, it is suggested that *Ef-cd* interacts with the histone modifier SDG724 to deposit H3K36me3 promoting transcription at *OsSOC1* (Fang et al. 2019). Ef-cd was identified as a major quantitative trait locus (QTL) for early flowering, where it was determined that disrupting Ef-cd, but not OsSOC1, led to delayed maturity. Thus, genetic and molecular data suggest that both *LAIR* and *Ef-cd* act as positive epigenetic regulators and reflect the potential for lncRNAs to impact agronomic traits.

### Regulating expression through promoter binding and ribonucleoprotein scaffolding

LncRNAs can also directly regulate transcription through targeted association with regulatory elements. One prominent example of this can be found in *HIDDEN TREASURE 1* (*HID1*). In response to constant red light, *HID1*, along with unknown protein partners, targets the promoter of *PHYTOCHROME INTERACTING FACTOR3* (*PIF3*), resulting in transcriptional inhibition of *PIF3* (Wang et al. 2014a). PIF3 is a transcription factor that inhibits developmental responses to red light and experiences rapid degradation by the light-activated phytochrome A and B photoreceptors (Al-Sady et al. 2006; Ni et al. 2013). In one of the most striking demonstrations of plant lncRNA conservation to date, the Arabidopsis *hid1* mutant phenotype was rescued using the *HID1* homolog from Rice, an evolutionary distance of ∼160 million years (Doyle 2012; Magallón et al. 2013). Indeed, sequence homologs of *HID1* have been identified in the moss, *Physcomitrium patens*. Interestingly, this sequence and functional conservation appear to be largely driven by two highly structured snoRNA-like domains, further blending the boundaries between snoRNAs and lncRNAs (Wang et al. 2014a).

Another example of lncRNAs targeting regulatory elements comes from the lncRNA *ELENA1* (*ELF18-INDUCED LONG-NONCODING RNA1*), which positively regulates plant pathogen defense. Specifically, *ELENA1* expression is induced by the bacterial pathogen-associated molecular patterns, elf18 and flg22 (Seo et al. 2017). *ELENA1* then directly interacts with and enriches the mediator subunit 19A (MED19A) at the distal promoter region of the *PATHOGENESIS-RELATED GENE1* (*PR1*) gene. *PR1* expression is induced and subsequently upregulates a suite of genes involved in the biotic stress response (Seo et al. 2017). In a follow-up study, Seo et al. (2019) further dissect this mechanism by showing that *ELENA1* removes FIBRILLARIN 2 (FIB2), a direct interactor of MED19A and transcriptional repressor, from the *PR1* promoter. Thus, pretranscriptional regulation of gene expression by lncRNAs can occur through a number of mechanisms, enhancing or repressing depending on context.

## Co- and posttranscriptional regulation of gene expression, mRNA abundance, and translation

### Influencing expression through transcriptional interference

LncRNAs can also regulate mRNA abundance and turnover after transcription has initiated, either co- or posttranscriptionally. Co-transcriptional regulation has been observed through the physical interference of Pol-II complexes when transcribing antisense or adjacent gene pairs (i.e. polymerase collision; Hobson et al. 2012). *SVALKA* and antisense-*CBF1* transcripts (*asCBF1*), lncRNAs located in the *C-repeat/dehydration-responsive element binding factors* (*CBFs*) gene cluster, function in this fashion (Kindgren et al. 2018). During the early cold stress response in Arabidopsis, rapid upregulation of the *CBF* gene cluster occurs (Medina et al. 1999, 2011). The CBFs are transcription factors that induce expression of the *COLD REGULATED* (*COR*) gene family (Fowler and Thomashow 2002). Activation of the *COR* genes results in swift biochemical and physiological changes allowing for freezing tolerance (Fowler and Thomashow 2002; Zhao et al. 2016). After several hours (>4) of cold exposure, transcription of an adjacent and antisense lncRNA to *CBF1*, *SVALKA*, begins. Transcription of *SVALKA* continues into the 3′ portion of the *CBF1* gene, generating unstable *asCBF1* through a phenomenon known as read-through transcription. This transcriptional read-through leads to stalling of the Pol-II complex transcribing the sense *CBF1*, resulting in attenuated expression of *CBF1* and tight control of the acclimation response. While we are only aware of one plant lncRNA currently described with this mechanism, the compact intergenic space of the Arabidopsis genome and the large number of lncRNAs residing in this space (Liu et al. 2012; Palos et al. 2022) suggest this mechanism may be more widespread.

### Influencing mRNA fate through alternative splicing and isoform selection

Alternative splicing has long been recognized as a critical mechanism impacting mRNA fate and function that can be dictated by lncRNAs. While much of our understanding of this lncRNA-guided mechanism comes from humans, there are now prominent examples of this in plants as well (Hutchinson et al. 2007; Clemson et al. 2009). The Arabidopsis lncRNA, *ASCO*, localizes to nuclear speckles (nuclear domains enriched in pre-mRNAs and splicing factors) and mediates alternative splicing of numerous target precursor mRNAs (Bardou et al. 2014). *ASCO* directly interacts with the spliceosomal subunits PRP8a (PRE-MRNA PROCESSING 8a) and SmD1b (Sm ring D1b) and mediates spliceosomal interactions with target mRNAs (Rigo et al. 2020). Loss or overexpression of *ASCO* leads to significant alternative splicing events across hundreds of transcripts. Initial studies showed that *ASCO* can mediate alternative splicing in roots, and assist in shifting isoform abundances to regulate cell fate during auxin-induced organogenesis (Bardou et al. 2014). However, more recent studies have shown that *ASCO* can also mediate the splicing of genes involved in plant biotic responses (Rigo et al. 2020). The role of *ASCO* in alternative splicing indirectly affects the function of hundreds of target transcripts, and thus the full influence, and other lncRNAs like it, remain unknown.

Alternative isoforms can be enriched through lncRNA-mediated co-transcriptional mechanisms. For example, an exon-derived long circular RNA (long-circRNA) transcribed from *SEPALLATA3* (*Sep3*) can impact the abundance of *SEP3* isoforms. *SEP3* encodes for a MADS-box transcription factor that is important for flowering time and floral organ identity (Liu et al. 2009). By forming a DNA–RNA hybrid, or R-loop, with the cognate locus, the *SEP3* long-circRNA encourages transcriptional pausing and effectively forces the locus to favor the production of alternative isoforms (Conn et al. 2017). Furthermore, the overexpression of the *SEP3* long-circRNA results in increased petal numbers and reduced stamen numbers through the promotion of alternative *SEP3* mRNA isoforms, suggesting that the *SEP3* long-circRNA is associated with proper floral organogenesis. Thus, the *SEP3* long-circRNA serves as the first example of a plant lncRNA mediating homeotic phenotypes via both R-looping and alternative splicing. In addition, *SEP3* represents the first mechanistic characterization of a circRNA in plants.

### LncRNAs that influence mRNA stability by sequestering miRNAs

At the posttranscriptional level, cytoplasmic-localized lncRNAs can influence gene expression by interacting with miRNA-mediated pathways and directly interacting with cytoplasmic proteins. *IPS1* (*induced by phosphate starvation 1*) and other noncoding transcripts within the *TPSI1/MT4* family described above (Burleigh and Harrison 1997; Liu et al. 1997; Chiou 2007) present another functional archetype of plant lncRNAs in modulating gene expression. *IPS1* was the first lncRNA, across all biology, to exhibit the capacity to sequester miRNA (Franco-Zorrilla et al. 2007). Under ambient conditions, *miRNA-399* acts to repress translation of its target mRNA—*PHO2*, a negative regulator of inorganic phosphate (Pi) accumulation (Lin et al. 2008). However, under phosphate starvation, *IPS1* inhibits *PHO2* degradation by sequestering *miRNA-399* away from *PHO2* mRNAs. Interestingly, *IPS1* evades miRNA-mediated degradation through incomplete sequence complementarity, creating a robust regulatory mechanism (Franco-Zorrilla et al. 2007). In this context, *IPS1* target mimicry allows for the fine-tuning of plant responses to Pi starvation by modulating the regulatory capacity of PHO2.

The functional characterization of *IPS1* and discovery of miRNA target mimicry often go understated. This discovery fueled new avenues of research and presented an entirely novel layer of gene regulation. One month after the *IPS1* result was published, a group working on mammalian miRNAs published a method to suppress miRNA action through transient expression of a RNA with miRNA binding sites (Ebert et al. 2007). This method was adapted in Arabidopsis to generate a collection of miRNA knockdown lines and examine their phenotypes (Todesco et al. 2010). During the preparation of this review, a group reported the mechanism of *lncRNA39896* which is involved in the tomato response to *Phytophthora infestans* infection (Hong et al. 2022). This lncRNA was previously predicted to be a target mimic for *miRNA-166b* based on sequence complementarity, expression characteristics, and degradome sequencing analysis (Cui et al. 2020). The *miRNA-166B* targets two mRNAs encoding homeodomain leuzine zipper transcription factors (HD-ZIP III), *SIHDZ34* and *SIHDZ45*, which are responsible for attenuating jasmonic acid and ethylene responses during biotic infection. The authors showed that *lncRNA39896* effectively decoys *miRNA-166B*, allowing translation of the target mRNAs and regulation of the hormone responses. Mutating the miRNA binding site of *lncRNA39896* led to increased miRNA targeting of the *SIHDZ34* and *SIHDZ45* mRNAs, a higher level of jasmonic acid and ethylene abundance, and increased resistance to Phytophthora infection. Thus, the discovery of endogenous target mimicry has contributed to our understanding of a gene regulatory mechanism with clear biotechnological applications.

### Impacting the last step of the central dogma: lncRNAs regulating translation

There are very few known instances of lncRNAs directly acting as regulators of mRNA translation. In rice, a cis-NAT of *PHOSPHATE1; 2* (*PHO1; 2*) is strongly upregulated during phosphate starvation and leads to the increase of PHO1; 2 protein levels (Jabnoune et al. 2013). Importantly, *PHO1; 2* mRNA levels remain stable across phosphate levels, as well as its isoform abundance and nuclear export patterns. The *PHO1; 2* cis-NAT achieves this increase in PHO1; 2 protein abundance through increased occupancy of both the cis-NAT and *PHO1; 2* mRNA occupancy at polysomes. Overexpression of the *PHO1; 2* NAT in trans leads to increased PHO1; 2 protein levels even in phosphate-replete conditions. Findings such as this have led to the development of technologies to activate translation through the expression of natural or synthetic long antisense RNAs (Zucchelli et al. 2015). In a separate global analysis of phosphate starvation stress, Bazin and colleagues performed ribosome footprinting to analyze noncoding RNA occupancy in roots shifted from phosphate replete to limited conditions (Bazin et al. 2017). They identify over 1,000 annotated lncRNAs with ribosome footprint signatures and nearly half of these lncRNAs are cis-NATs. These data suggest a potentially widespread and largely unexplored mechanism of translational regulation akin to the *PHO1; 2* cis-NAT discussed above. As the number of translatome datasets increase (e.g. ribo-seq, polysome profiling, etc.) in plants, they will allow researchers to distinguish between novel small ORF containing transcripts and those that regulate translation in a manner similar to the cis-NAT of *PHO1; 2*, and perhaps identify novel cytoplasmic lncRNA functions outside of translational regulation away from the ribosome.

## Plant lncRNAs outside of regulating gene expression

While a majority of the mechanistically described plant lncRNAs govern every aspect of the progression from DNA to protein, there are several lncRNAs that function outside of this role. As large, multidomain molecules with complex structures, lncRNAs are ideal platforms on which other molecules (proteins or RNA) can dock. This innate ability to interact with other molecules has been alluded to above in the context of gene expression, but also occurs in other contexts in both the nucleus and the cytoplasm.

Perhaps the most famous of these lncRNAs is the *TR*, the RNA component of a ribonucleoprotein complex called telomerase which is essential for maintaining chromosome ends. Despite its characterization in ciliates, yeast, and vertebrates in the 20th century (Greider and Blackburn 1989; Singer and Gottschling 1994; Feng et al. 1995), *TR* was only recently characterized in plants (Fajkus et al. 2019; Song et al. 2019; Dew-Budd et al. 2020). In each of these lineages, *TR* serves as a scaffold for the binding of the reverse transcriptase, TERT, as well as a number of accessory proteins critical for complex maturation and function. This scaffolding function is dependent upon conserved structures within *TR*, however, *TRs* are highly divergent in both their sequences and biogenesis pathways, suggesting the acquisition of novel *TRs* into the telomerase complex. Excitingly, plant genomes have a preponderance of *TR* paralogs and additional TERT interacting RNAs (Nelson and Shippen 2015), suggesting that plants may have more lessons to teach in terms of lncRNA-mediated regulation and the relaxed parameters under which even functionally conserved lncRNAs evolve.

Another example of a scaffolding RNA with a putative role in ribosome assembly and biogenesis is *lncCOBRA1* (*CONSERVED IN BRASSICA RAPA 1*; Kramer et al. 2022). As the name suggests, this lncRNA was identified through an examination of Arabidopsis nuclear lncRNAs which were protein bound and conserved across Brassicaceae (Gosai et al. 2015). Mutant *lnccobra1* plants show delayed germination and generally grow slower than wild-type. *lncCOBRA1* seems to act through scaffolding of various protein partners, particularly RACK1A, which is important for ribosome assembly and biogenesis (Guo et al. 2011). Finally, it is notable that *lncCOBRA1*, similar to *HID1*, is a polycistronic transcript containing two highly conserved snoRNAs. Although these snoRNA domains do not appear to be further processed, they are highly structured and display high sequence conservation rates across Brassicaceae relative to the rest of the *lncCOBRA1* locus. In addition, these two domains were reported as the targets for protein binding (Gosai et al. 2015; Qu et al. 2015). Thus, *lncCOBRA1* and *HID1* represent scaffolding lncRNAs that impact Arabidopsis development and were identified through comparative and molecular signatures.

Another important RNA–protein interaction can be seen in the first identified plant lncRNA, *ENOD40*. In *M. truncatula*, the *ENOD40* RNA interacts with a pre-mRNA splicing factor RBP1 (RNA BINDING PROTEIN1), a nuclear speckle RNA-binding protein (Campalans et al. 2004). In non-nodulating plant cells, RBP1 is nuclear localized. However, during root nodulation, *ENOD40* and RBP1 translocate to the cytoplasm and this translocation is dependent on *ENOD40* expression. Thus, *ENOD40* appears to act in a similar manner to *ASCO* through the regulation of nuclear speckle splicing factors. However, in this case, *ENOD40* is acting as both an environmental sensor and a guide, facilitating the relocalization of its target protein.

In addition to aiding in protein localization, lncRNAs can also compete for protein interaction partners, with profound impacts on plant development. One of the early lncRNA identification efforts in rice (Zhang et al. 2014) led to the identification of *MISSEN*, a cytoplasmically localized lncRNA that regulates endosperm development (Zhou et al. 2021). *MISSEN* was identified based on its high expression levels in young flowers and pistils. Disruption of the *MISSEN* locus by T-DNA insertional mutagenesis led to decreased seed set, misshapen seeds, and reduced endosperm size. Mechanistically, *MISSEN* acts by competitively inhibiting a helicase family protein from interacting with tubulin, subsequently impacting endosperm development (Zhou et al. 2021). Finally, *MISSEN* was shown to be specifically expressed from the maternal allele during endosperm development, a feature common during endosperm and seed development (Huh et al. 2007). Given the contexts of expression, *MISSEN* provides a model for how regulatory properties of lncRNAs can affect fundamental stages of plant development.

## Novel approaches for inferring function

De novo structural identification and classification of lncRNAs in a transcriptomic and genomic context provides the foundation to explore the functional roles of plant lncRNAs. However, prioritizing lncRNAs for functional analysis is not as straightforward as for protein-coding genes. Functional and comparative resources (e.g. RNA functional domain databases) are lacking, restricting the development of functional clues when a lncRNA is identified in a researcher's RNA-seq data. While we expect functional annotation to become easier as more lncRNAs are described, there are currently only a few bioinformatic methods for assigning putative functions to lncRNAs, including integrating lncRNAs into co-expression and gene regulatory networks as well as motif enrichment analyses that we will describe below.

### Guilt-by-association

To better understand the function of lncRNAs, clustering lncRNAs with highly correlated expression to protein-coding genes using weighted gene co-expression networks, often referred to as “Guilt-by-Association” (GBA), is gaining traction in both plants and animals (Langfelder and Horvath 2008; Liu et al. 2022; Nolte et al. 2022; Waseem et al. 2022; Zhang et al. 2022a). Given similarities in expression profiles across a complex dataset, genes will group together into modules. Gene ontology term and other similar pathway analysis databases (e.g., Kyoto Encyclopedia of Genes and Geomes or KEGG) can identify the protein-associated biological processes enriched within that module. The GBA comes from the assumption that lncRNAs with statistically similar expression patterns will likely be involved in similar processes as proteins within a module. Overlaying additional information, such as transcription factor binding data (e.g. coming from DNA affinity purification sequencing or DAP-seq; O’Malley et al. 2016; Bartlett et al. 2017), can further constrain these modules and assist in inferring the directionality of interactions. Ultimately, these types of analyses help researchers build testable functional hypotheses.

When paired with additional types of data, GBA approaches can be quite informative and help pinpoint functional lncRNAs. For instance, a functional annotation of chromatin-enriched ncRNAs in rice incorporated expression, chromatin-interaction data, and phenotypic information to identify lncRNAs associated with yield (Zhang et al. 2022b). In addition, Palos et al. (2022) identified germination-associated lncRNAs using a GBA approach through the incorporation of expression data from the Klepikova tissue atlas (Klepikova et al. 2016). The authors identified a module of co-expressed genes with peak expression in embryogenesis and germination. A further phenotypic screen of mutants in lncRNAs found within this module revealed reduced germination rates. These approaches benefit from experimental complexity, be it temporal, tissue, or treatment based, as well as pairing with complementary epigenetic, structural, or interaction datasets. Under these circumstances, GBA approaches can be incredibly useful in helping researchers identify when and where to look for function.

### Identification of functional domains

LncRNA functional domains, as with protein domains, are often the regions of the RNA through which intermolecular interactions (RNA–protein, RNA–RNA, and RNA-metabolite) occur. These domains, or motifs, are often short (6 to 12 nt) and may occur multiple times within the same RNA to increase binding affinity or number of interactions (Ulitsky et al. 2011; Bitetti et al. 2018). As a result, those motifs, and their enrichment, may serve as signatures of function and can even help uncover patterns of conservation. Sequence homology-based approaches of Brassicaceae lncRNAs revealed higher levels of conservation within regions found to be structured and protein bound (Nelson et al. 2016; Palos et al. 2022). More elegant comparative analyses of vertebrate lncRNAs revealed a higher degree of conservation within functional motifs as compared to nonmotif fragments within the same RNA, indicating some level of selection was occurring (Hezroni et al. 2015; Ross and Ulitsky 2022). These approaches can further help identify orthologous lncRNAs in the absence of overall sequence similarity. One example of this comes from the LncLOOM framework (Lncrna Linear Order cOnserved Motifs) which searches for retention in the order of multiple short motifs within the same lncRNA to determine homology and ultimately functional conservation (Ross et al. 2021). LncLOOM was used in vertebrates to identify orthologs of the vertebrate-conserved lncRNA, *CYRANO* (Ulitsky et al. 2011). The *Cyrano* gene family was originally identified due to transcription arising from a syntenic locus. However, despite the identification of homologs across vertebrates, *Cyrano* is highly sequence and length divergent and it was difficult to infer functional orthology. Upon deeper inspection of the *Cyrano* gene family with LncLOOM, seven functional motifs, always arranged in the same order, were uncovered within this lncRNA across vertebrates. Syntenic, but sequence-divergent lncRNAs have also been observed in plants (Palos et al. 2022), and LncLOOM, or similar approaches, may help uncover conserved functional domains and guide in their evolutionary analyses.

Innovations in natural language processing and machine learning methods have made it possible to use functional motifs from characterized lncRNAs to infer function of unknown lncRNAs. In this context, algorithms such as SEquence Evaluation from K-mer Representation (SEEKR) search for the enrichment of particular motifs, or k-mers, within a lncRNA (Kirk et al. 2018). K-mer signatures are developed for a particular query lncRNA and then pairwise Pearson-correlation is used to search for other lncRNAs with similar k-mer signatures. SEEKR was successfully used to identify two human lncRNAs (*KCNQ1OT1* and *AIRN*) that exhibited similar k-mer profiles to the *XIST* lncRNA, a well-known lncRNA involved in the epigenetic silencing of the x chromosome through interactions with the polycomb repressor complex (Zhao et al. 2008). Further analysis revealed that the observed correlation between k-mer signatures was driven by PRC interaction domains found in each of the lncRNAs. Indeed, through a clustering analysis, SEEKR identified hundreds of lncRNAs with similar k-mer signatures as *XIST*, suggesting a large set of PRC-interacting epigenetic regulators may exist in mammals, an observation further bolstered by an abundance of genome-wide PRC-RNA interaction data. Thus, k-mer or motif-based lncRNA analyses may help to predict lncRNA function. One caveat to the SEEKR and LncLOOM approaches outlined above is that they were both developed predominantly in mammalian systems and thus may require retooling for plant lncRNA functional motifs (SEEKR) and plant genome evolution (LncLOOM). However, in silico functional predictions and inferences of orthology are likely to only get stronger as more interaction partners and functional domains are identified for plant lncRNAs.

### High-throughput genetic screens for perturbing lncRNA expression

In addition to bioinformatic approaches, an improved molecular toolkit for plant systems would greatly facilitate the identification of functional lncRNAs. CRISPR/Cas9 (clustered regularly interspaced short palindromic repeats) gene editing approaches have already demonstrated their utility in plants for assessing gene function in a highly targeted fashion (Zhou et al. 2021; Kramer et al. 2022). However, lncRNAs lack many of the fundamental features typically used as targets by CRISPR approaches (i.e. disrupting ORFs through frame-shift mutations). CRISPR-mediated gene or promoter deletions are options, and are certainly ideal over standard insertional mutagenesis approaches long used in Arabidopsis (e.g. T-DNA insertional mutagenesis). Indeed, CRISPR-mediated lncRNA deletion was used to ascertain the function of at least two lncRNAs, including *lncCOBRA1* and *FLAIL* (Jin et al. 2021b; Kramer et al. 2022). However, targeted deletion of both lncRNA loci or their promoters is largely guesswork, particularly in the absence of well-annotated transcription start site information, and is not particularly high-throughput as a functional screen. A promising CRISPR-based alternative for screening for functional lncRNAs can be found in CRISPR activation/inactivation systems (CRISPRi/a) that were recently developed for mammalian systems (Jensen et al. 2021) and are starting to gain traction in plants (Gilbert et al. 2013, 2014; Liu et al. 2017). The CRISPRi/a systems work by fuzing either repressive or activating effector domains to Cas9 and then using guide RNAs designed to the approximate transcriptional start site of a lncRNA of interest to effectively modulate expression. This type of system can be multiplexed with the addition of 100s–1000s of guide RNAs to screen through a suite of target lncRNAs (Liu et al. 2017; Covarrubias et al. 2020). This technology is in the early stages in plants, but has successfully been applied in a few protein-centric manners (Lowder et al. 2017; Ochoa-Fernandez et al. 2020; Kar et al. 2022) and will undoubtedly have an outsized impact on lncRNA biology.

## Future functional forays: understudied aspects of plant lncRNAs

### Localization

A lncRNA's subcellular localization can inform in which functional archetype the gene may be involved (Fig. 2). Nuclear lncRNAs may perform a variety of pre or cotranscriptional modes of gene regulation, genome stability, or mediating chromosomal interactions (Guh et al. 2020; Li et al. 2021; Xiao et al. 2022). In contrast, cytoplasmic lncRNAs might scaffold proteins, decoy miRNAs, regulate translation, or a variety of other less understood lncRNA actions (Noh et al. 2018). Multiple studies have investigated lncRNAs from nuclear and/or cytoplasmic compartments (Zhao et al. 2018; Do et al. 2019), and there are emerging computational approaches to predict lncRNA subcellular localization (Su et al. 2018; Ahmad et al. 2020). These datasets represent valuable resources to the plant lncRNA community as they present the foundation for follow-up molecular experiments; integration of such datasets to a central repository or database of plant lncRNAs (e.g. STRING for lncRNAs; Jensen et al. 2008) would bolster and accelerate all aspects of plant RNA biology.

One avenue of lncRNA subcellular localization that remains untouched in plants is their role in the formation of SGs. SGs are cytoplasmic protein, RNA, and metabolite aggregates that form in response to stress conditions, and are thought to serve as crucial reservoirs of ribosomal machinery, chaperones, and untranslated mRNAs (Kearly et al. 2022). SGs are a conserved phenomenon across all eukaryotes, and in mammals, lncRNAs play some role in their formation, potentially through disordered domains or through their scaffolding abilities (Khong et al. 2017; Campos-Melo et al. 2021; Maruri-López et al. 2021). This likely holds true in plants and would highlight another important cytoplasmic function for lncRNAs.

### Mobilization

In addition to subcellular localization, the role of cell–cell mobile lncRNAs is another exciting new avenue for plant lncRNA research. There is ample evidence that the plant vasculature serves as a long-distance communication system for transporting proteins, metabolites, hormones, as well as RNAs (Morris 2018; Thomas and Frank 2019). Indeed, one group has analyzed the lncRNA repertoires that show movement through the phloem during phosphate deficiency in cucumber (Zhang et al. 2019). They identified hundreds of mobile lncRNAs that are responsive to phosphate deficiency, including the cucumber homolog to *IPS1* and other putative miRNA mimic lncRNAs. In support of this finding, Thieme et al. (2015) identified an Arabidopsis paralog of *IPS1* (*AT4*) in a pool of mobile mRNAs. Furthermore, these mobile lncRNAs may be important for interspecies communication. A recent study demonstrated that a green peach aphid (*Myzus persicae*) lncRNA (*Ya1*) is part of a pool of molecules injected into the aphid's plant host (Arabidopsis) and that this particular lncRNA promotes aphid feeding (Chen et al. 2020). Interestingly, *Ya1* was observed to migrate from the aphid feeding site to other leaves, perhaps interacting with a target molecule in those leaves to reduce plant defenses. Thus, lncRNA mobility, within plants, and between organisms, is an important aspect to consider when predicting targets and molecular mechanisms.

### The role of alternative splicing in regulating lncRNA function

Alternative splicing is an important mechanism for delivering regulatory plasticity in response to changing cellular or organismal environments by allowing for multiple RNAs from the same locus (Syed et al. 2012; Reddy et al. 2013). The scale and regulation of alternative splicing in plant protein-coding premessenger RNAs is well documented; over 60% of multiexonic genes undergo alternative splicing (AS) in plants (Chaudhary et al. 2019). While most plant lncRNAs appear to be mono-exonic (∼90% in Arabidopsis; Nelson et al. 2016; Palos et al. 2022), a large number of the multiexonic Arabidopsis lncRNAs display splice site conservation across Brassicaceae (Corona-Gomez et al. 2020). In addition, AS has been well-described for certain lncRNAs, such as *COOLAIR* and *FLORE* (Csorba et al. 2014; Henriques et al. 2017). The biological role of AS in plant transcripts is unclear, as many mRNA isoforms are rapidly degraded by the nonsense-mediated decay machinery (Filichkin et al. 2015). Noncoding, alternative isoforms of protein-coding genes may represent a novel form of posttranscriptional gene regulation, as they have been found to influence mRNA levels arising from the same locus (Reddy 2007). While the mechanistic reason behind lncRNA AS is not as immediately clear as it is for mRNAs, AS may allow for alternative targeting (for cis or trans-regulatory lncRNAs), or for alternative protein–RNA interactions that then facilitate unique functions. Thus, AS is an exciting aspect of plant lncRNAs that requires further exploration.

### Regulating the regulator

While lncRNAs have broadly become established as regulators of gene expression, there is substantially less known regarding how lncRNAs themselves are regulated and how this feeds back into their function. Genome-wide assessment of DNA methylation and histone modification profiles in multiple plant species has demonstrated that lincRNA loci closely resemble protein-coding loci. In particular, lincRNAs in Arabidopsis and *Eutrema salsugineum* displayed enrichment of H3K4me3 (trimethylation of lysine 4 on histone 3) near the promoter and 5′ start site along with H3K36me3 (trimethylation of lysine 36 on histone 3) across the gene body and flanking both ends of the transcript start and end sites (Zhang et al. 2009; Heo et al. 2013; Palos et al. 2022). This local connection of H3K4me3 and H3K36me3, also referred to as the K4-K36 domain, is associated with actively transcribed protein-coding genes and was used to identify numerous deeply conserved, highly expressed, and multiexonic lincRNAs in humans and mouse (Guttman et al. 2009; Khalil et al. 2009). Less is known about the relationship between DNA methylation and lncRNA expression, but from the limited studies to date, the relationship between expression and DNA methylation is generally negative, particularly in the gene body (Zhou et al. 2021; Palos et al. 2022; Yu et al. 2022). Much more work is needed to better understand how the epigenome modulates lncRNA expression.

Once the chromatin is relaxed, transcriptional machinery needs to be recruited in order to initiate lncRNA transcription. Interestingly, distinct differences have been observed in this regard in Arabidopsis. Tokizawa et al. (2017) mapped transcription start sites and characterized the promoters and capping characteristics of coding and noncoding genes. They observed that antisense lncRNA and lincRNA promoters have lower ratios of TATA boxes and Y patches (pyrimidine patches) that are generally necessary for transcription. This relative depletion of TATA boxes has been noted in animals for antisense lncRNAs (Lin et al. 2015). These findings suggest a potential explanation for the low abundance of lncRNAs as a class: if lncRNAs contain nonoptimal promoters, Pol-II may be less efficiently recruited in the absence of additional factors. Another intriguing finding was that only ∼42% of antisense lncRNAs and ∼74% of lincRNAs were associated with CAGE-generated capped transcription start sites, potentially suggesting rapid turnover or alternative biogenesis pathways (Tokizawa et al. 2017).

RNA biogenesis pathways are another underexplored aspect of lncRNA regulation and turnover. Work from Zhi John Lu's group (as mentioned above in the sampling bias section) has shed light upon lncRNAs that do not contain polyA tails, a facet of lncRNA research that is underexplored. For instance, Yuan et al. (2018) showed that while hundreds of Rice lncRNAs are downregulated during abiotic stress, isoforms of these lncRNAs that do not contain polyA tails actually increase in abundance (Yuan et al. 2018). One possibility is that the abundance of these lncRNAs are modulated by the exosome in a manner similar to *ASL* in Arabidopsis (Shin and Chekanova 2014). The biological implications and reasoning for this phenomenon are not understood, yet it presents exciting opportunities for uncovering even more complex modes of lncRNA-involved transcriptional regulation, as these RNAs may be overlooked in standard sequencing approaches.

### Understanding the impact of the epitranscriptome on lncRNAs

Finally, every aspect of an RNA's lifecycle, including its shape, interacting partners, and ultimately function, can be influenced by chemical base modifications often referred to as the epitranscriptome (Bhatia et al. 2022). Aside from the 5′ trimethylguanosine cap and recently identified alternatives (Wang et al. 2019b; Yu et al. 2021), there are a number of RNA base modifications known to impact mRNA structure and function in plants (Shen et al. 2019). While a more exhaustive review of the epitranscriptome can be found elsewhere in this issue, we believe that these modifications are likely also widespread on lncRNAs and have just been overlooked due to the general low abundance and poor annotation of lncRNAs. However, targeted studies have demonstrated that the epitranscriptome is just as critical for lncRNAs as it is for mRNAs and sRNAs. For instance, N6-methyladenosine was recently shown to be important for *COOLAIR's* role in regulating the *FLC* locus by modulating R-loop stability (Xu et al. 2021). Given COOLAIR's ability to adopt multiple structural conformations that impact flowering (Yang et al. 2022), considering both RNA modifications and structure in tandem will be critical for dissecting mechanism. Global analyses of RNA modifications in Arabidopsis using the bioinformatic tool HAMR found distinct patterns between stable lncRNAs and those targeted for degradation, suggesting modification state may be indicative of the functional state (Ryvkin et al. 2013; Vandivier et al. 2015). Single-molecule techniques capable of directly identifying modifications on RNAs, such as Oxford Nanopore's direct RNA-sequencing approach (Kirov et al. 2020), are poised to dramatically alter our understanding of the epitranscriptome in plants. In sum, the impact of the epitranscriptome on lncRNA biology in plants is an emerging field with the potential to explain how lncRNAs function at substoichiometric levels, how they recruit or interact with binding partners, and ultimately how they impact plant biology.

## Conclusions with an eye towards the future of plant lncRNA biology

Tremendous achievements have been made over the last 25 yr in plant lncRNA biology. Characteristics and mechanisms of lncRNAs first discovered in nonmodel species, then elaborated on in Arabidopsis, are now being used to identify functionally important lncRNAs across the plant lineage. However, at present, the function of the vast majority of annotated plant lncRNAs remains unknown, and traditional molecular investigations into each one of these transcripts would be cost-prohibitive. We anticipate modern-omics technology, in combination with rapid CRISPR-based genetic screening and ML approaches, will close the gap between putative and actual functions for plant lncRNAs.

Discoveries in a few key plant species will undoubtedly translate to more and more distant relatives as we begin to understand the rules that govern lncRNA evolution. Moving away from a protein-centric perspective will be critical. Selective pressures at the nucleotide, or even structural level, are likely less important for some lncRNA functions than others. In addition, expression abundance—typically thought of as a sign of functionality in protein-coding genes—may be less important than expression at the right or optimal time or arising from the correct portion of a genome. Developing comparative frameworks that incorporate these types of conservation will be critical in transferring functional information from one species to another.

New and exciting tools and techniques are being brought to bear on the identification, functional dissection, and comparative analyses of lncRNAs. As computational algorithms and approaches are specifically developed for lncRNAs, robust sets of functional lncRNAs will be needed for training. Critical thought will need to be placed into which lncRNAs are selected as positive controls to avoid training around non-lncRNA features. To aid in algorithm development, the current plant lncRNA databases need to be harmonized in terms of the criteria each used to identify their lncRNAs.

In all of this, the variation in functional archetypes is important to keep in mind. While many lncRNAs likely influence the genome and transcriptome in the nucleus, there is growing evidence that cytoplasmic functions are just as varied. The ability of lncRNAs, alongside other molecules, to translocate between cells or organisms further deepens the mystery of what role these enigmatic transcripts play in plant biology. The functional possibilities seem endless, and the chance for mistaken identities is high, but the long and storied history of lncRNAs in plants makes it clear that their study is worth the effort.

## Data Availability

No data were generated in the preparation of this review.
